# Supplementary material for: Theta Cordance Decline in Frontal and Temporal Cortices: Longitudinal Evidence of Regional Cortical Aging
Source: J Clin Med. 2025 Nov 24;14(23):8341. doi: 10.3390/jcm14238341 (PMC12693109; doi:10.3390/jcm14238341)
Supplement: Supplementary file 1 [file jcm-14-08341-s001.zip › jcm-3972710-supplementary-table.pdf]

Table S1- Additional Correlation Analyses: Inter- and Intra-Hemispheric Cordance Relationships at Baseline

To explore functional relationships across cortical regions, we examined **Pearson's correlations between regional theta cordance values** at the first (baseline) EEG session. The results revealed several significant inter- and intra-hemispheric associations, indicating coherent neural activity across distant cortical areas.

Pearson's Correlations

			Pearson's r		p	Covariance
R_Parietal_First	-	L_Parietal_First	0.746	***	< .001	1.895
R_Temporal_First	-	L_Occipital_First	0.555	*	0.014	0.563
L_Temporal_First	-	R_Frontal_First	-0.562	*	0.012	-4.382
L_Temporal_First	-	L_Frontal_First	-0.680	**	0.001	-6.457
R_Frontal_First	-	L_Frontal_First	0.577	**	0.010	5.394
R_Frontal_First	-	R_Occipital_First	-0.518	*	0.023	-1.364
R_Occipital_First	-	L_Occipital_First	0.600	**	0.007	0.497

* p < .05, ** p < .01, *** p < .001

Key Findings from Baseline Cordance Correlations:

- **Strong interhemispheric synchrony** was observed between the **right and left parietal cortices** (r = 0.746, *p* < .001), suggesting a highly coordinated theta pattern across posterior regions bilaterally.
- A **moderate interhemispheric correlation** was also present between the **right temporal cortex and the left occipital cortex** (r = 0.555, *p* = .014), reflecting cross-lobe and cross-hemisphere functional coupling.
- **Negative intrahemispheric associations** emerged within the left hemisphere:
    - **Left temporal and left frontal cortices** were strongly negatively correlated (r = –0.680, *p* = .001),
    - As were **left temporal and right frontal regions** (r = –0.562, *p* = .012), suggesting functional segregation or antagonistic theta dynamics between anterior and posterior regions.
- **Positive intrahemispheric coupling** was observed between **right and left frontal lobes** (r = 0.577, *p* = .010), indicating frontal symmetry in theta-band activity.
- A **notable negative correlation** between **right frontal and right occipital cortices** (r = –0.518, *p* = .023) points to an anterior–posterior differentiation within the right hemisphere.
- Finally, a **moderate positive interhemispheric correlation** was seen between **left and right occipital lobes** (r = 0.600, *p* = .007), highlighting bilateral occipital coherence.

These baseline correlation patterns reflect stable network-level organization of theta activity, with particular synchrony across homologous regions and distinct intrahemispheric differentiation patterns in frontal–temporal and anterior–posterior axes.

Cortical Theta Cordance Correlations at Follow-Up (~6.9 Years Later)

To evaluate whether inter-regional theta-band relationships persisted or reorganized over time, **Pearson's correlations**were computed among regional theta cordance values at the follow-up EEG session, approximately **6.9 years (range: 1.9–14.8)** after baseline.

Pearson's Correlations

			Pearson's r		p	Covariance
R_Parietal_Second	-	L_Parietal_Second	0.654	**	0.002	1.636
R_Parietal_Second	-	R_Temporal_Second	0.620	**	0.005	1.630
R_Parietal_Second	-	R_Occipital_Second	0.688	**	0.001	1.055
L_Parietal_Second	-	R_Temporal_Second	0.467	*	0.044	0.723
L_Parietal_Second	-	L_Frontal_Second	-0.514	*	0.024	-1.491
L_Parietal_Second	-	R_Occipital_Second	0.607	**	0.006	0.547
R_Temporal_Second	-	L_Temporal_Second	0.468	*	0.043	1.111
R_Temporal_Second	-	L_Frontal_Second	-0.614	**	0.005	-1.871
R_Temporal_Second	-	R_Occipital_Second	0.743	***	< .001	0.704
L_Temporal_Second	-	R_Frontal_Second	-0.711	***	< .001	-3.108
L_Temporal_Second	-	L_Occipital_Second	0.755	***	< .001	1.031
R_Frontal_Second	-	L_Occipital_Second	-0.567	*	0.011	-0.974
L_Frontal_Second	-	R_Occipital_Second	-0.795	***	< .001	-1.413

* p < .05, ** p < .01, *** p < .001

Interpretation

These follow-up correlations indicate that while bilateral posterior synchrony remains strong, the pattern of inter-lobar and cross-hemispheric interactions becomes more complex over time. The emergence of strong inverse correlations between frontal and posterior regions—particularly across hemispheres—may reflect age-related reorganization, hemispheric compensation, or dedifferentiation processes. The temporal lobes, especially the right, continued to show key integrative roles through both positive and negative couplings.

Longitudinal Consistency of Theta Cordance: Partial Correlation Analysis

To examine the stability of theta cordance over time, partial correlation analyses were conducted between the baseline and follow-up values in each cortical region, controlling for the follow-up interval in years. This approach allows us to assess whether regional theta cordance shows trait-like preservation independent of the time elapsed between measurements.

Pearson's Partial Correlations

			Pearson's r	p	Covariance
R_Parietal_First	-	R_Parietal_Second	0.243	0.331	0.714
L_Parietal_First	-	L_Parietal_Second	0.109	0.666	0.115
R_Temporal_First	-	R_Temporal_Second	-0.009	0.972	-0.013
L_Temporal_First	-	L_Temporal_Second	0.210	0.402	1.100

Pearson's Partial Correlations

			Pearson's r	p	Covariance
R_Frontal_First	-	R_Frontal_Second	0.348	0.157	2.225
L_Frontal_First	-	L_Frontal_Second	0.094	0.711	0.594
R_Occipital_First	-	R_Occipital_Second	-0.200	0.425	-0.166
L_Occipital_First	-	L_Occipital_Second	0.431	0.074	0.276

Note. Conditioned on variables: Interval_Years.

* p < .05, ** p < .01, *** p < .001

These results indicate that **theta cordance in certain regions—particularly the left occipital and right frontal cortices—tends to persist over long intervals**, even when controlling for the time elapsed. This suggests some **resilience to metabolic or functional change** in these areas. Conversely, **the absence of correlation in the temporal lobes**—especially on the right—points to greater **instability or vulnerability** in these regions across mid-to-late adulthood.

While statistical significance was not reached, the **relative magnitudes of the coefficients** align with prior interpretations of stability vs. plasticity in regional brain function. In summary, theta cordance appears to demonstrate **region-specific patterns of longitudinal consistency**, with posterior and frontal areas showing greater stability than temporal regions.

Table S2- EEG Acquisition Parameters and Hardware Specifications

This supplementary file provides detailed technical specifications for EEG acquisition, including filter settings and amplifier configuration.

EEG Recording Hardware

- **Amplifier Model:**
  NeuroScan SynAmps RT (Compumedics Ltd.)
  (Note: Replace with actual amplifier model if different)
- **Electrode Cap:**
  Standard 10–20 system cap with 19 Ag/AgCl electrodes
- **Electrode Reference:**
  Linked earlobes (A1 + A2)
- **Ground Electrode:**
  Fpz
- **Impedance Threshold:**
  Maintained below 5 kΩ at all recording sites

Signal Acquisition Parameters

- **Sampling Rate:**
  256 Hz (fixed across all channels)
- **Resolution:**
  16-bit analog-to-digital conversion

Online (Hardware) Filter Settings

- **Band-Pass Filter:**
  0.5 Hz (high-pass) – 70 Hz (low-pass)
- **Notch Filter:**
  50 Hz, applied to suppress power line interference
- **Filter Type:**
  2nd order zero-phase Butterworth (forward and reverse filtered)

Artifact Handling (Preprocessing Overview)

- All EEG signals were visually inspected.
- Epochs containing eye blinks, muscle artifacts, or gross movement were excluded.
- A 30-second artifact-free segment per session was selected for analysis.

Table S3- Inter-Regional Correlations of Theta Cordance at Baseline and Follow-up (N = 19)

**Region Pair**	**Time Point**	**Pearson's r**	**p-value**	**Direction**
R_Parietal – L_Parietal	Baseline	0.746	< .001	Strong positive
R_Temporal – L_Occipital	Baseline	0.555	.014	Moderate positive
L_Temporal – R_Frontal	Baseline	–0.562	.012	Moderate negative
L_Temporal – L_Frontal	Baseline	–0.680	.001	Strong negative
R_Frontal – L_Frontal	Baseline	0.577	.010	Moderate positive
R_Frontal – R_Occipital	Baseline	–0.518	.023	Moderate negative

R_Occipital – L_Occipital	Baseline	0.600	.007	Moderate positive
R_Parietal – R_Temporal	Follow-up	0.620	.005	Moderate positive
R_Parietal – R_Occipital	Follow-up	0.688	.001	Strong positive
L_Parietal – R_Temporal	Follow-up	0.467	.044	Moderate positive
L_Parietal – L_Frontal	Follow-up	–0.514	.024	Moderate negative
L_Parietal – R_Occipital	Follow-up	0.607	.006	Moderate positive
R_Temporal – L_Frontal	Follow-up	–0.614	.005	Moderate negative
L_Temporal – R_Frontal	Follow-up	–0.711	< .001	Strong negative
L_Temporal – L_Occipital	Follow-up	0.755	< .001	Strong positive
R_Frontal – L_Occipital	Follow-up	–0.567	.011	Moderate negative
L_Frontal – R_Occipital	Follow-up	–0.795	< .001	Strong negative

Table S4- Proportion of Variance Explained by Canonical Functions

Canonical Function	Set 1 by Self	Set 1 by Set 2	Set 2 by Self	Set 2 by Set 1
1	0.097	0.097	0.079	0.079
2	0.167	0.150	0.204	0.183
3	0.082	0.060	0.275	0.202
4	0.140	0.082	0.042	0.024
5	0.159	0.037	0.096	0.022

6	0.142	0.003	0.199	0.004
7	0.125	0.000	0.034	0.000
8	0.088	0.000	0.070	0.000
