# Supplementary material for: Theta Cordance Decline in Frontal and Temporal Cortices: Longitudinal Evidence of Regional Cortical Aging
Source: J Clin Med. 2025 Nov 24;14(23):8341. doi: 10.3390/jcm14238341 (PMC12693109; doi:10.3390/jcm14238341)

Tarih : 11.12.2018

THETA KORDANS SONUÇLARI

Hastanın Adı soyadı :

**Prefrontal (Fp1,FzFp2)**	: -1,699
**Frontosantral (F3,F4,Fz)**	: -0,896
**Santral (C3, Cz,C4)**	: 1,094
**Sol temporal (T3,T5)**	: 2,625
**Sağ temporal (T4,T6)**	: -0,029
**Sol paryetal (P3,T5)**	: 1,234
**Sağ paryetal(P4,T6)**	: -0,494
**Oksipital (O1,O2)**	: 0,341

**C3**	2,133
**CZ**	0,167
**C4**	0,981
**P3**	0,715
**P4**	-0,106
**T4**	0,825
**F3**	0,017
**FZ**	-0,796
**F4**	-1,907
**F8**	-1,172
**F7**	0,476
**T3**	3,497
**FP1**	-1,479
**FP2**	-2,822
**O1**	0,712
**O2**	-0,030
**T5**	1,753
**T6**	-0,882
**PZ**	-2,081

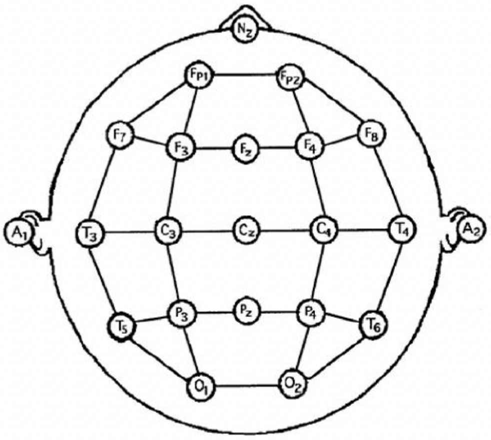

Supplement: Supplementary file 1 [file jcm-14-08341-s001.zip › jcm-3972710-supplementary.pdf]
